# Evaluation of the cervical liquid-based cytology sample as a microbiome resource for dual diagnosis

**DOI:** 10.1371/journal.pone.0308985

**Published:** 2024-12-11

**Authors:** Chiwoong Lim, Young-Jun Seo, Ji-Yeong Lee, Eun Sung Jung, Sunyoung Lee, Hayoung Kim, Kidong Kim, Jun-Mo Kim

**Affiliations:** 1 Department of Animal Science and Technology, Chung-Ang University, Anseong-si, Gyeonggi-do, Republic of Korea; 2 HEM Pharma Inc., Suwon, Gyeonggi, Republic of Korea; 3 Ildong Bioscience, Pyeongtaek, Republic of Korea; 4 Department of Obstetrics and Gynecology, Seoul National University Bundang Hospital, Seongnam, Republic of Korea; 5 Department of Obstetrics and Gynecology, Seoul National University College of Medicine, Seoul, Republic of Korea; Universidad San Francisco de Quito, ECUADOR

## Abstract

Cervical cancer, which is mainly caused by oncogenic human papillomavirus subtypes, remains a significant global health challenge. Recent studies have indicated a connection between cervical cancer and the uterine microbiome, underscoring its importance. This study explored the potential of liquid-based cytology (LBC) samples, which are typically used for cytological analysis, in investigating the cervical microbiome. Thirty women participated in the study and provided clinical information. Three samples were obtained from each participant: one for clinical purposes using LBC, another for microbiome sampling using LBC, and a third using the SWAB Microbiome kit. The LBC and traditional swab (SWAB) samples were subjected to high-throughput 16S rRNA gene sequencing for microbiome analysis. The results revealed a consistent dominance of key taxa, particularly *Lactobacillus* spp. The analysis of differential abundance highlighted variations in microbial abundance among individuals, which were more prominent than those resulting from the sampling methods. Functional analysis identified arachidonic acid and alpha-linolenic acid metabolism, along with a cautionary note regarding the low mean proportion values. The network analysis revealed positive correlations between indicators of structure among the networks, highlighting the robustness of microbiome similarities despite the diversity of sampling methods. Supervised machine learning has revealed challenges in distinguishing LBC and SWAB samples based on their microbiome features. Weighted co-expression network analysis revealed that the correlation between microbial clusters and the sampling method with clinical data was not significant. This study emphasizes the similarity in microbial communities observed using the LBC and SWAB methods, highlighting the potential of using dual diagnostic approaches. Additionally, the use of residual LBC samples in large-scale microbiological studies can provide comprehensive insights into cervical health and disease.

## Introduction

Cervical cancer remains a significant global health challenge despite advances in its prevention and early detection through screening programs that target pre-invasive lesions, known as cervical intraepithelial neoplasia (CIN) [1]. The main cause of both CIN and cervical cancer is persistent infection with oncogenic subtypes of the human papillomavirus (HPV) [2, 3]. Although HPV infections are highly prevalent, with a lifetime risk exceeding 80%, the progression to CIN and cervical cancer is a protracted process, often taking several years to decades [4].

Recent studies have revealed the potential interplay between the cervical microbiome and disease development in HPV-positive women, revealing greater bacterial diversity than their HPV-negative counterparts [5, 6]. Cervical microbiota has been identified as a crucial factor influencing the progression of cervical CIN and is implicated in the persistence of high-risk and low-risk HPV [7]. This indicates a complex relationship in which the cervical microbiome influences the interaction between HPV infection and the development of cervical pathology [8]. Certain bacterial taxa, such as Fusobacteria and Sneathia, are strongly associated with HPV infection [9]. They also facilitate or modify viral entry, replication, or immune evasion. Conversely, vaginal flora, dominated by *Lactobacillus* spp, has been identified as a protective factor against various pathogens, including HPV [10]. Furthermore, changes in the composition of the cervical microbiome have been observed during HPV infection, indicating a interaction between the viral infection and the microbial community that affects disease progression [10]. This interplay can impact the development of the disease, potentially altering the shift from initial infection to pre-invasive lesions, and ultimately leading to cervical cancer [11].

Despite these findings, the potential use of the cervical microbiome for cervical cancer screening and diagnosis remains an area of active investigation [12]. Liquid-based cytology (LBC) is a method of collecting and preserving cervical cells in an alcohol-based fixative [13]. LBC offers distinct advantages over conventional methods, such as the Pap smear, providing improved sample preservation and ease of examination. Despite these advantages, the potential use of LBC samples for cervicovaginal microbiome studies remains largely unexplored [14]. Using high-throughput sequencing of 16S rRNA gene amplicon sequences, studies have provided valuable insights into the relationship between cervicovaginal microbiota and HPV infection, as well as HPV-related diseases [15]. This technology is a powerful tool for characterizing microbial communities and provides insight into their role in cervical health and disease progression. The integration of 16S rRNA sequencing into the analysis of LBC samples has the potential to enhance microbiome characteristics in cervical cancer [16].

Although conventional microbiome studies often rely on swabs or self-collected vaginal discharge samples, few studies have focused on the utility of LBC samples [17]. This study aimed to explore the potential of LBC samples as a resource for microbiome analysis. In addition, we suggest a potential resource for assessing the feasibility of utilizing LBC samples for cervical microbiome surveys, with the ultimate objective of improving the ability to diagnose multiple conditions related to cervical cancer.

## Materials and methods

### Participants, clinical data, and sample collection

The study population was selected from women aged 19 and above who visited the Obstetrics and Gynecology department at Seoul National University Bundang Hospital and were going to receive medically indicated Pap smear within one year of Institutional Review Board (IRB) approval (B-2011-651-301) (from January 18, 2021, to February 2, 2021) and performed in accordance with the principles of the Declaration of Helsinki. The study’s ethical considerations were emphasized through additional explanation sessions before obtaining participant signatures, aiming to minimize any potential for coercion or undue influence. A total of 30 participants were recruited and ensure a voluntary and informed participation process, eligible individuals were provided with a detailed explanation of the study’s objectives, methods, potential benefits, and risks by the responsible investigator and written informed consent was obtained from all participants. Sample collection involved the use of BD Surepath collection kits (Becton, Dickinson and Company, 1 Becton Drive, Franklin Lakes, NJ 07417–1880) for both medically indicated and research-specific liquid-based cervical cytology. Sterile speculum examination without lubricant a swab was first taken from the ectocervix using a T-SWAB TRANSPORTTM containing liquid Amies with a rayon tip (Noble Bio, Hwaseong, Korea). A total of three samples were collected from one participant, including two LBC-based samples for medical diagnostic and research purposes and one sample collected using a rayon tip. Clinical information, including age, height, body weight, menopausal status, medication history, vaginal douching practices, sexual intercourse, and Pap smear results was systematically recorded for each participant.

### Microbial DNA extraction, library construction, sequencing, and pre-processing

Bacterial genomic DNA extraction was performed using Mag-Bind® Universal Pathogen Kit (Omega Bio-t, Norcross, Georgia). Each liquid sample was cell-downed, the pellet was resuspended in 1X PBS and then mixed with 275 μL of SLX-Mlus Buffer, followed by bead beating in a mixermill MM400 (Retsch, Pennsylvania, USA) with further isolating, cleaning, and eluting procedures followed the manufacturer’s protocols. Sequencing libraries were prepared according to the Illumina 16S Metagenomic Sequencing Library protocols (Illumina, San Diego, CA, USA) to amplify the V3 and V4 regions. The input genomic DNA (gDNA; minimum 20 ng) was polymerase chain reaction (PCR)-amplified with2× KAPA HiFi HotStart ReadyMix (Roche, Basel, Switzerland) and DNA were generated by PCR under conditions of 3 min at 95°C, followed by 25 cycles at 95°C for 30 s, annealing at 55°C for 30 s, extension at 72°C for 30 s and a final extension at 72°C for 5 min. The universal primer pair with Illumina adapter overhang sequences used for the first set of amplification was as follows: V3-F: 5′-TCGTCGGCAGCGTCAGATGTGTATAAGAGACAGCCTACGGGNGGCWGCAG-3′; V4-R: 5′-GTCTCGTGGGCTCGGAGATGTGTATAAGAGACAGGACTACHVGGGTATCTAATCC-3′. The first PCR product was purified using HiAccuBead (AccuGene, Incheon, Korea). Following purification, 2 μl of the first PCR product was PCR-amplified for final library construction using the IDT indexing primer (Integrated DNA technologies). The cycle conditions for the second PCR were the same as those for the first PCR condition except for 10 cycles. The PCR products were purified using HiAccuBead (AccuGene, Incheon, Korea). The final purified product was quantified by Qubit 4.0 (ThermoFisher Scientific, Waltham, MA USA) with 1× dsDNA HS assay solution (ThermoFisher Scientific, Waltham, MA USA). Paired-end (2 × 250 bp) sequencing was performed by HEM Pharma lnc. using the MiSeq platform (Illumina, San Diego, CA, USA).

Low-quality reads of the original data were filtered using Cutadapt (version 1.9.1) [18], and the data from each sample were separated by barcodes. The barcode and primer sequences were then cut off to obtain raw reads, which were further aligned with the Gold database (version 1) using the UCHIME algorithm to remove chimeric sequences and obtain clean reads [19].

Representative operational taxonomic units (OTUs) (high frequency) were screened and annotated according to the SILVA (Latin Silva, Forest, http://www.arb-silva.de) small subunit rRNA database. Subsequently, MUSCLE software (version 3.8.31) [20] was used to perform multiple sequence alignments of the OTUs to determine their phylogenetic relationships. OTU abundances were normalized using standard sequence numbers corresponding to the sample with the least number of sequences, based on which diversity analysis was performed. Within-sample alpha diversity was calculated according to the genus profile to determine microbial communities.

### Characterization of LBC and traditional swab samples (SWAB) microbiome

Alpha diversity analysis, richness (observed amplicon sequence variants), and Shannon diversity indices [21] were estimated using Quantitative Insights into Microbial Ecology (QIIME2) core-metrics-phylogenetic and alpha-group-significance scripts [22]. Beta diversity was estimated by calculating unweighted and weighted UniFrac distances and then visualized using principal coordinate analysis (PCoA) and non-metric multidimensional scaling. Linear discriminant analysis coupled with effect size was performed to identify the bacterial taxa that were differentially represented at different taxonomic levels [23]. Categorization of the amplicon sequence variants was performed based on a 97% similarity threshold using the SILVA (version 132) reference database, resulting in the formation of OTUs. Representative sequences were assigned to OTUs using a naïve Bayes classifier (SILVA, v132) trained specifically for the 16S rRNA V3–V4 hypervariable region. The classification was performed using the q2-feature-classifier plugin.

This analysis was performed using QIIME2 software with core-metrics-phylogenetic and Microbiota Process [24]. The overall microbial composition was determined at the genus level. Following the conversion of all taxonomic count data to relative abundance, a colored bar plot was generated to display all abundant taxonomic groups at the genus level for each individual. The four samples with the highest overall sequence quality were selected for further investigation, as determined using a ranking system based on quality scores. Subsequently, the Statistical Analysis of Metagenomic Profiles (STAMP) program was used to analyze the microbial correlation between LBC and SWAB samples [25]. Pearson’s correlation coefficients were calculated to evaluate the associations among microbial taxa, and statistical significance was determined by incorporating multiple testing correction methods. The resulting correlation analyses were visualized using STAMP graphical capabilities, aiding in interpreting the strength and direction of associations between microbiomes in LBC and SWAB samples.

### Comparative functional analysis of microbiome

To address the challenges associated with zero issues in microbiome analysis, we implemented a robust methodology that considers recent advancements and best practices. Given the limitations of the pseudo-counts, particularly their deviation from the negative binomial distribution assumption of DESeq2, alternative strategies were explored. A recent update in DESeq2 introduced novel estimators within the estimateSizeFactors function, facilitating accurate size factor determination even when samples contain zeros for all genes. Additionally, we opted to use alternative size-factor estimators rather than transformed counts in the DESeq2 analysis. To further enhance the reliability of our analysis, we applied a filtering step to exclude features with more than 90% zero occurrences across all samples, mitigating the impact of sparse features on DESeq2 modeling. To visualize the identified biomarkers, we used the R package ComplexHeatmap, an advanced tool built on a pheatmap. Although these alternative packages adhere to the same heatmap theory, we specifically utilized ComplexHeatmap::pheatmap for its inheritance of most settings from pheatmap, ensuring code transparency and ease of interpretation. Based on the predictive functional analysis results using PICRUSt2, differentially abundant pathways were identified using Welch’s t-test with STAMP software. This approach allowed for a rigorous examination of microbial functional profiles, considering the potential heterogeneity in variance between groups. Pathways with P values below the predefined significance threshold of 0.05 were selected as differentially represented. STAMP facilitated the comparison of the mean proportions of functional abundances between the two study groups, using a t-test for robust statistical assessment. The resulting differentially abundant pathways were further visualized using informative plots, providing insights into the predicted microbial functional variations across the experimental conditions.

### Construction of microbial networks

Microbial association network analysis was conducted using the SParse InversE Covariance Estimation for Ecological Association Inference (SPIEC-EASI), a robust method widely employed in microbiota investigations. Two distinct sets of association network analyses were performed to comprehensively explore the microbial interactions. Microbiota data across methods within an individual were aggregated to assess overall microbial associations within individuals. Before the analysis, the abundance data underwent centered-log ratio transformation, and OTUs with fewer than 50 and 10 reads were excluded from the first and second analyses, respectively. The Stability Approach to Regularization Selection method, suitable for high-dimensional data, was employed to infer the network structure. Utilizing the node-based neighborhood selection procedure, with a minimum lambda ratio of 0.01 and 50 reiterations, the Stability Approach to Regularization Selection facilitated the robust determination of the network architecture. The resulting networks were visualized using Cytoscape (version 3.4.0). General network structural attributes such as degree distribution and natural connectivity were determined for each network. Natural connectivity, a proxy for network stability, was assessed based on the removal of decreasing node betweenness centrality or decreasing node degree. Additionally, a subgraph correlation distance approach was applied to a set of networks to compare the roles of individuals in shaping the association network structure. This involved breaking down each network into subgraphs, generating a graphlet correlation matrix, and visualizing the relationships through multidimensional scaling plots constructed using R. The proximity of the networks in the plot space indicates similarity in the network structure.

### Supervised multivariate analysis via machine learning approach

Supervised multivariate analysis was conducted using the multivariate approach of sparse Partial Least Squares Discriminant Analysis (sPLS-DA), an extension of the PLS algorithm. The sPLS-DA model was further adapted for microbiome data using either CSS-normalized or TSS+CLR data within the mixMC framework. PLS-Discriminant Analysis is a multivariate regression model that optimizes the covariance between linear combinations of OTU counts and the outcome, represented by a dummy matrix indicating the body site of each sample. The sparse version, sPLS-DA, incorporates lasso penalizations for feature selection and applies component-wise to highlight the discriminative features in the model. Parameter optimization and performance evaluation were conducted through a 10-fold cross-validation repeated 100 times, enabling the determination of the optimal number of features and components for the sPLS-DA model. The final sPLS-DA model was executed on the entire dataset to obtain a definitive list of discriminative OTUs for each component. Graphical and numerical outputs were generated to characterize the selected OTUs, including bar plots illustrating the contribution of each feature in the multivariate model and circular representations of taxonomic trees using GraPhlAn. The contribution plots displayed bacterial taxonomy at the family level, with bar lengths indicating feature importance and colors corresponding to the contributing body sites. Additional outputs included sample representation, displaying individual projections onto the sPLS-DA components, a list of selected OTU features, cross-validation error rates, and the number of features contributing to the methods for each component. The multilevel sPLS-DA framework was implemented using the mixOmics R package, incorporating multilevel decomposition for enhanced microbiome data analysis.

### Weighted Co-Expression Network Analysis (WGCNA) of phenotype-microbiome interactions

Phenotype-microbiome interactions were investigated through WGCNS using the WGCNA R package. The analytical workflow comprised several sequential steps to uncover the intricate relationships between phenotypic traits and microbial genera. A correlation matrix was constructed by computing Pearson’s correlation coefficients for all pairs of phenotype-genera interactions. Subsequently, an adjacency matrix was generated based on the formula a_*mn*_ = |c_*mn*_|^*β*^, where a_*mn*_ represents the adjacency between phenotype/genus *m* and phenotype/genus *n*, c_*mn*_ denotes the Pearson’s correlation coefficient, and *β* is a soft-power threshold determined to achieve a standard scale-free topology network. The adjacency matrix was further transformed into a topological overlap matrix that captured the similarity in terms of the commonality of the connected nodes. The tree-cutting algorithm was then applied to hierarchically cluster the topological overlap matrix into modules, representing clusters of highly interconnected phenotypic traits and microbial genera. The parameters for the algorithm were set to minModuleSize = 5 for both the phenotype/genus dendrograms, ensuring a minimum module size and minimum height = 0.25 to cut the tree, facilitating the merger of similar modules. This meticulous approach aimed to reveal cohesive and functionally relevant associations between phenotypic traits and microbial genera, thereby providing insights into the complex interplay within the phenotype-microbiome landscape.

## Results

### Alpha & beta diversity analysis

**Fig 1:**
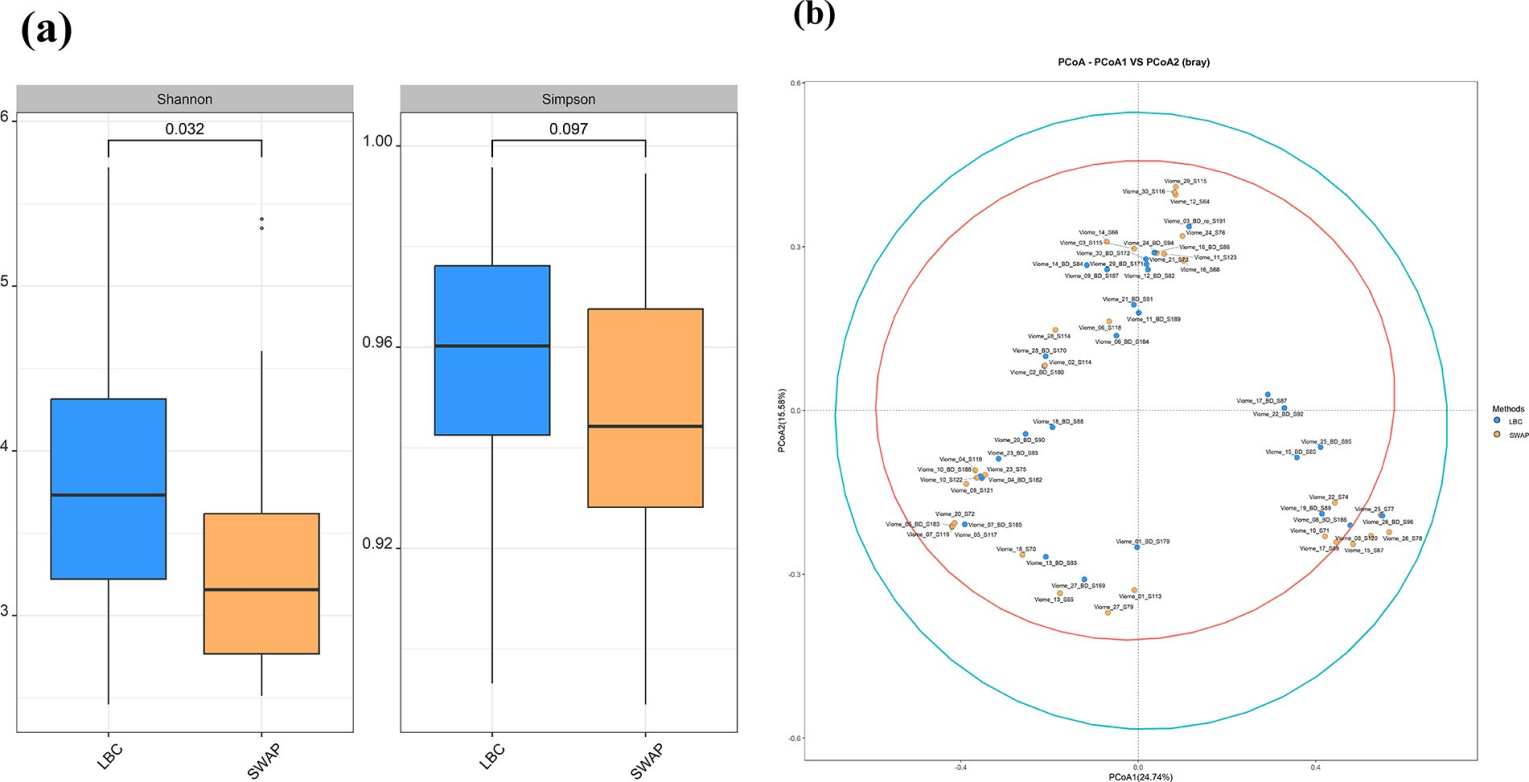
Diversity analysis of LBC and SWAB microbial composition. (a) Alpha diversity indexes (Shannon and Simpson) of the microbiota in the LBC and SWAB groups. (b) Bray-Curtis distance methods of bacterial 16S rRNA genes. Clustering indicated microbiota composition in LBC and SWAB.

The mean Shannon value, which measures species richness and evenness, was 3.72 in the LBC group and 3.18 in the SWAB group. Statistical analysis using the t-test revealed a significant difference between the two groups (p = 0.032). The mean Simpson diversity index of the LBC group was 0.974, whereas that of the SWAB group was slightly lower (0.9426). The t-test yielded a p-value of 0.097 (Fig 1A). Beta diversity analysis was conducted using the Bray-Curtis method to compare the microbial composition between samples. The PCoA analysis showed that the first principal component accounted for 27.74% of the variability, and the second principal component accounted for 15.58%. The results did not indicate a statistically significant difference in the overall cluster structure between the two sampling methods (p = 0.99, PERMANOVA) (Fig 1B).

### Taxonomic classifications and correlation analysis

**Fig 2:**
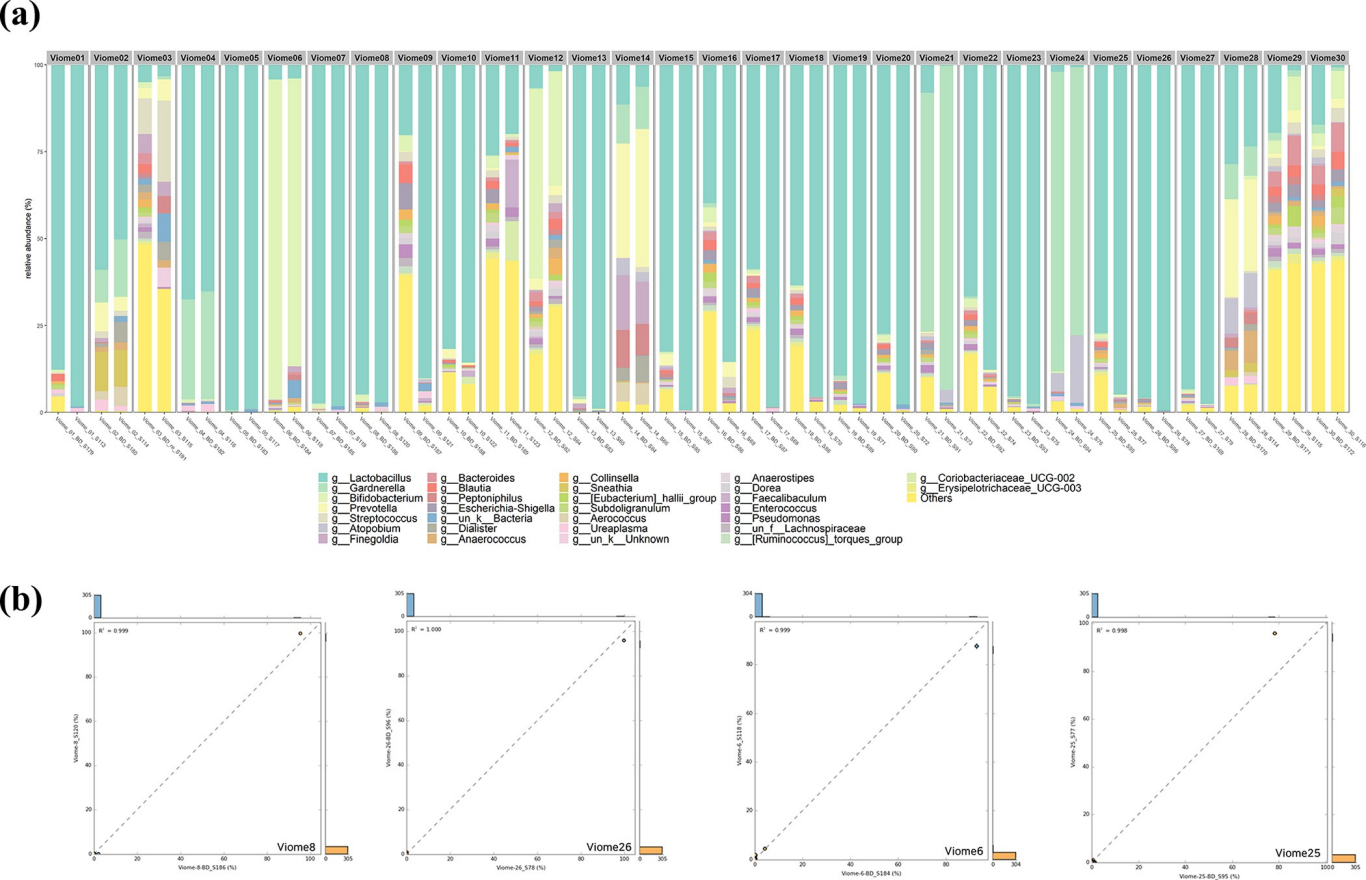
Taxonomic classifications and correlation analysis of LBC and SWAB microbiomes. (a) Relative abundance of LBC and SWAB microbiomes at the genus level. (b) Using a 2-group comparison of the correlation between compositions at the genus level.

Taxonomic classification at the genus level was conducted on the microbiome data derived from cervical LBC and SWAB samples (Fig 2A). In the order of abundance, the bacteria identified were *Lactobacillus*, *Gardnerella*, *Bifidobacterium*, *Prevotella*, and *Streptococcus* spp. Of the 25 samples, a correlation coefficient exceeding 0.9 was observed, indicating a consistent microbial signature across both sampling techniques (S1 Fig). Furthermore, four individuals with the highest sequencing quality was conducted, focusing on the correlation analysis of the top 10 microorganisms based on abundance (Fig 2B). The correlation coefficients for Viome8, Viome26, Viome6, and Viome25 were 0.999, 1.000, 0.999, and 0.998, respectively. Lactobacillus was identified as the most abundant genus among the top 20 microorganisms in Viome8 and included the highest-quality 16S rRNA sequence (S2 Fig). This genus accounted for more than 95% of the total abundance.

### Hierarchical clustering and functional enrichment analysis of differential abundance microbiome

**Fig 3:**
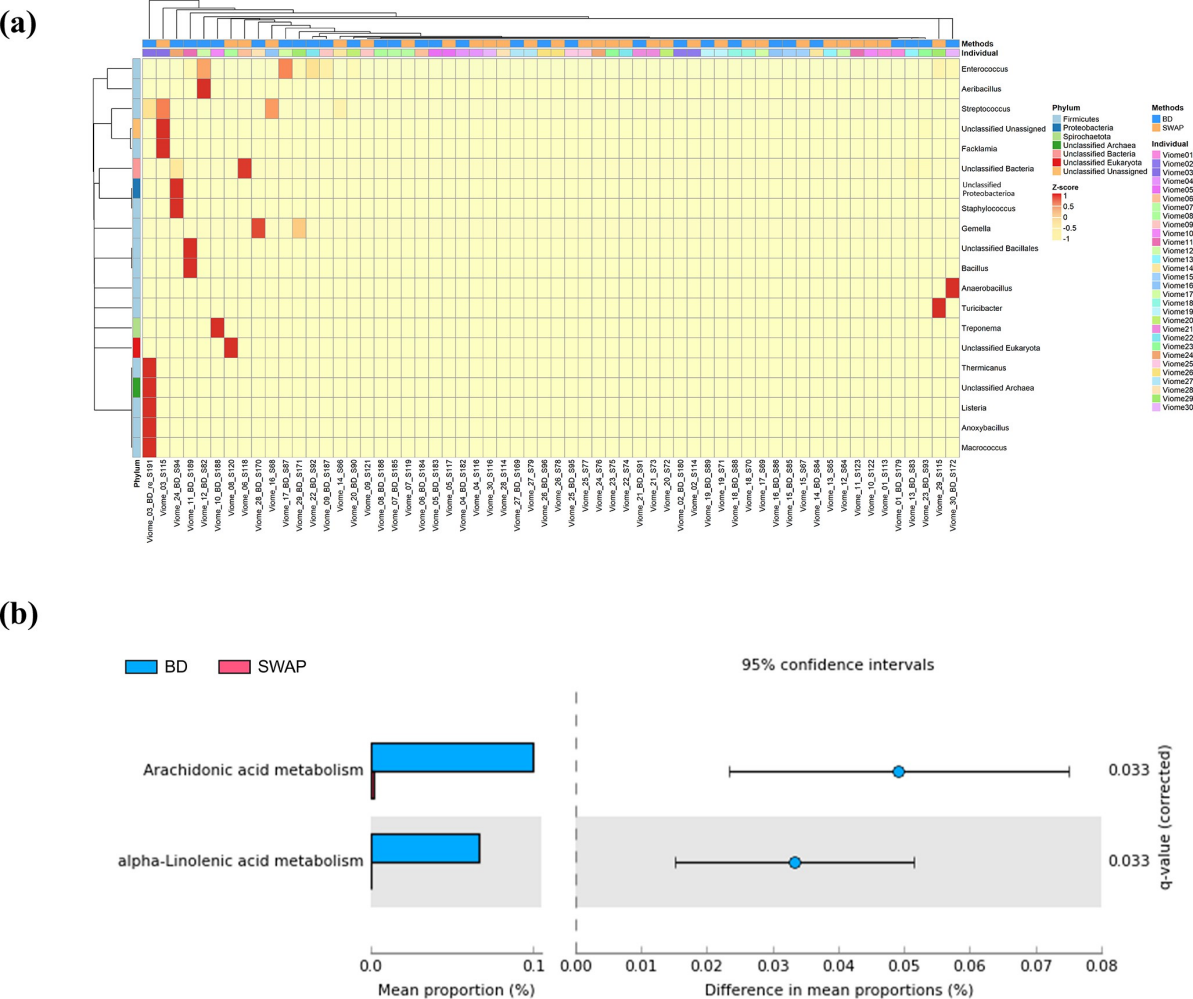
Differential abundance and functional enrichment analysis of LBC and SWAB microbiomes. (a) Hierarchical clustering was performed on 60 samples of microorganisms with significance at the genus and phylum levels. (b) Extended bar plot in STAMP, illustrating the abundance of biological function via the KEGG pathway.

The differential abundance analysis revealed 20 bacteria with significant differences at the genus level across the sampled cervical microbiomes. Hierarchical clustering at the phylum level was subsequently conducted, and the annotation process identified 17 of the 20 selected microorganisms (Fig 3A). Hierarchical clustering was performed on 60 samples of microorganisms showing significance at the genus level. Functional enrichment analysis revealed that the two Kyoto Encyclopedia of Genes and Genomes (KEGG) pathways exhibited statistical significance regarding microbial communities, with a p-value threshold of < 0.05, as determined using a t-test (Fig 3B). Functional analysis revealed arachidonic acid and alpha-linolenic acid metabolism, with both functional analyses showing significance levels of 0.033. In addition, the mean proportion values were high in LBC, with values of 0.1 and 0.07, and the difference in mean proportions showed that arachidonic acid metabolism was 0.051 and alpha-linolenic acid metabolism was 0.033.

### Comparison of network of LBC and SWAB samples

**Fig 4:**
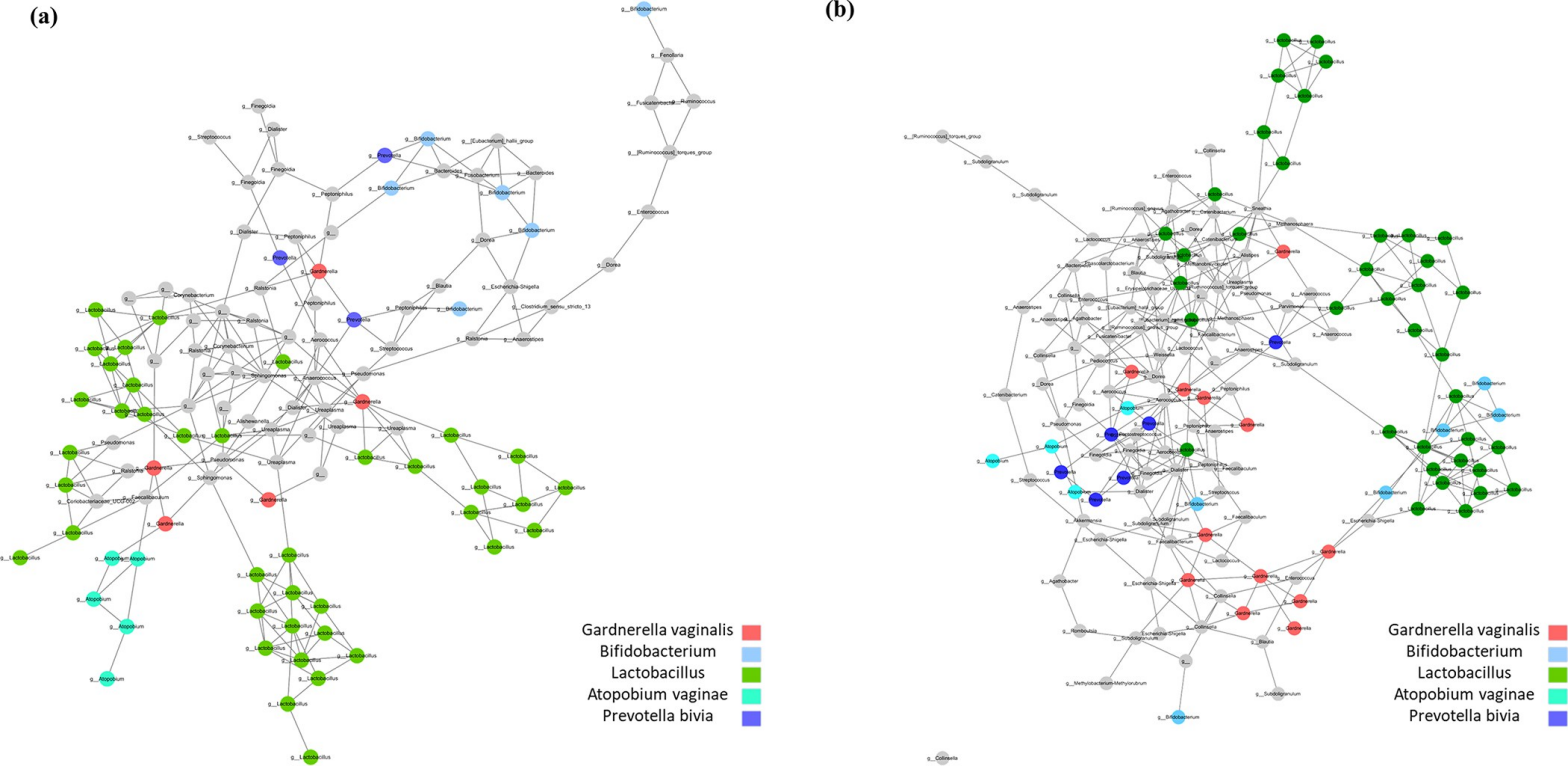
Characterization of microbiome co-occurrence network according to sampling methods. (a,b) Co-occurrence network of LBC and SWAB microbiome in order from the right. Each node represents a microbiome, and the edges of positively correlated nodes are connected. All nodes are labeled using the genus level taxonomic classification.

Co-occurrence patterns based on the cervical microbiome were analyzed using SPIEC-EASI (Fig 4A and 4B). The network structure showed only a positive correlation between the bacterial taxon communities, consistent with the co-occurrence principle inherent in the SPIEC-EASI algorithm.

To further analyze the structural aspects of the microbial network, several key network parameters were calculated. The total number of network nodes differed between the LBC and SWAB methods, with 158 and 155 nodes, respectively. The network density, which represents the ratio of the realized to the possible number of edges, was computed as 0.03 for SWAB and 0.023 for LBC. Additionally, the network heterogeneity, which indicates the tendency of a network to contain hub nodes, was 0.516 and 0.462 for SWAB and LBC, respectively. Finally, the average number of neighbors, which indicates the average number of edges per node, was 2.236 for SWAB and 2.452 for LBC.

### Sparse partial least squares discriminant analysis

**Fig 5:**
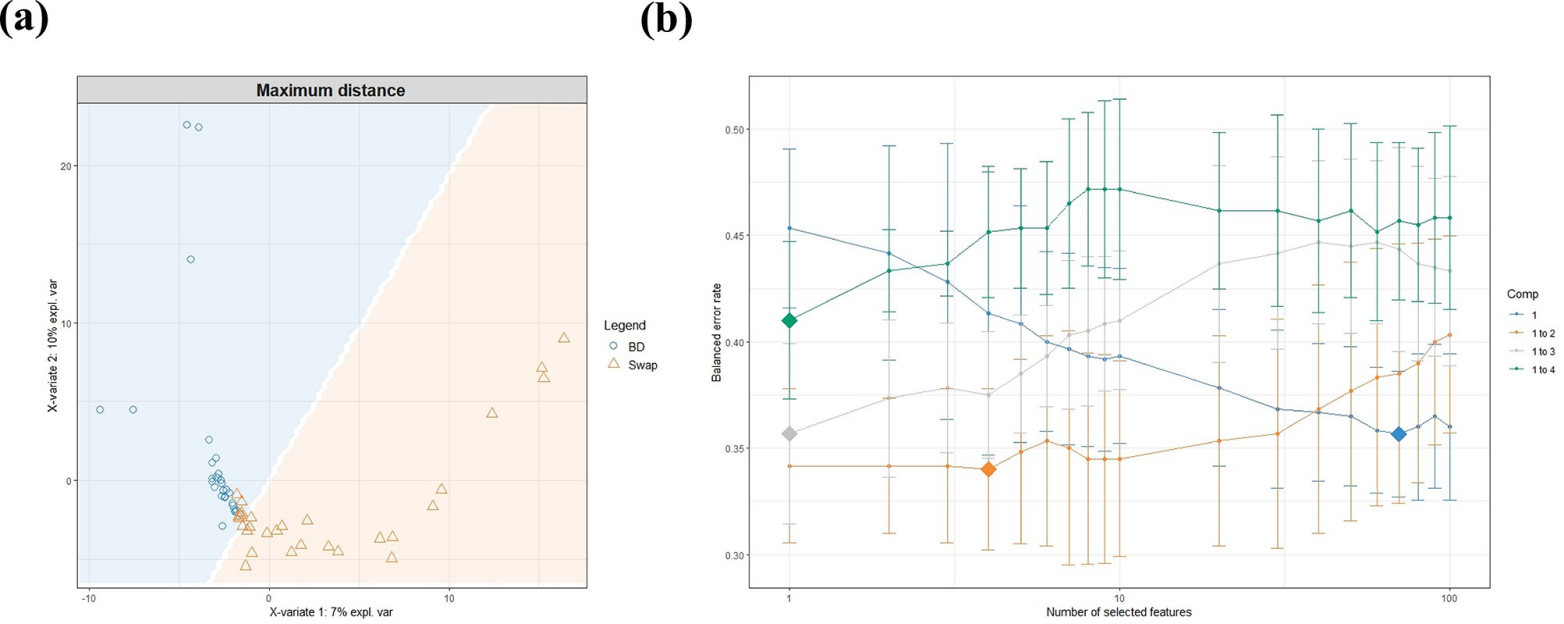
Sparse Partial Least Squares Discriminant Analysis (sPLS-DA) profiles for LBC and SWAB microbiomes. (a) sPLS-DA distances of bacterial 16S rRNA genes. Clustering indicated differences in microbiota composition between LBC and SWAB (b) Tuning keepX for the sPLS-DA performed on the microbiome data. Each colored line represents the balanced error rate (y-axis) per component across all tested keepX values (x-axis) with the standard deviation based on the repeated cross-validation folds.

Using supervised machine learning methods, the explanatory powers of the first and second variables were found to be 7% and 10%, respectively, when analyzed through maximum distance methods (Fig 5A). A distinct boundary was observed, separating the two methods (LBC and SWAB), with 35 samples represented at this border. Upon further analysis by selecting features that differed between the two methods, an initial error rate of 36% was recorded. As the main components were progressively added, there was a notable increase in the error rate, with the numbers escalating to 39%, 43%, and subsequently 47% (Fig 5B).

### WGCNA based on clinical data

**Fig 6:**
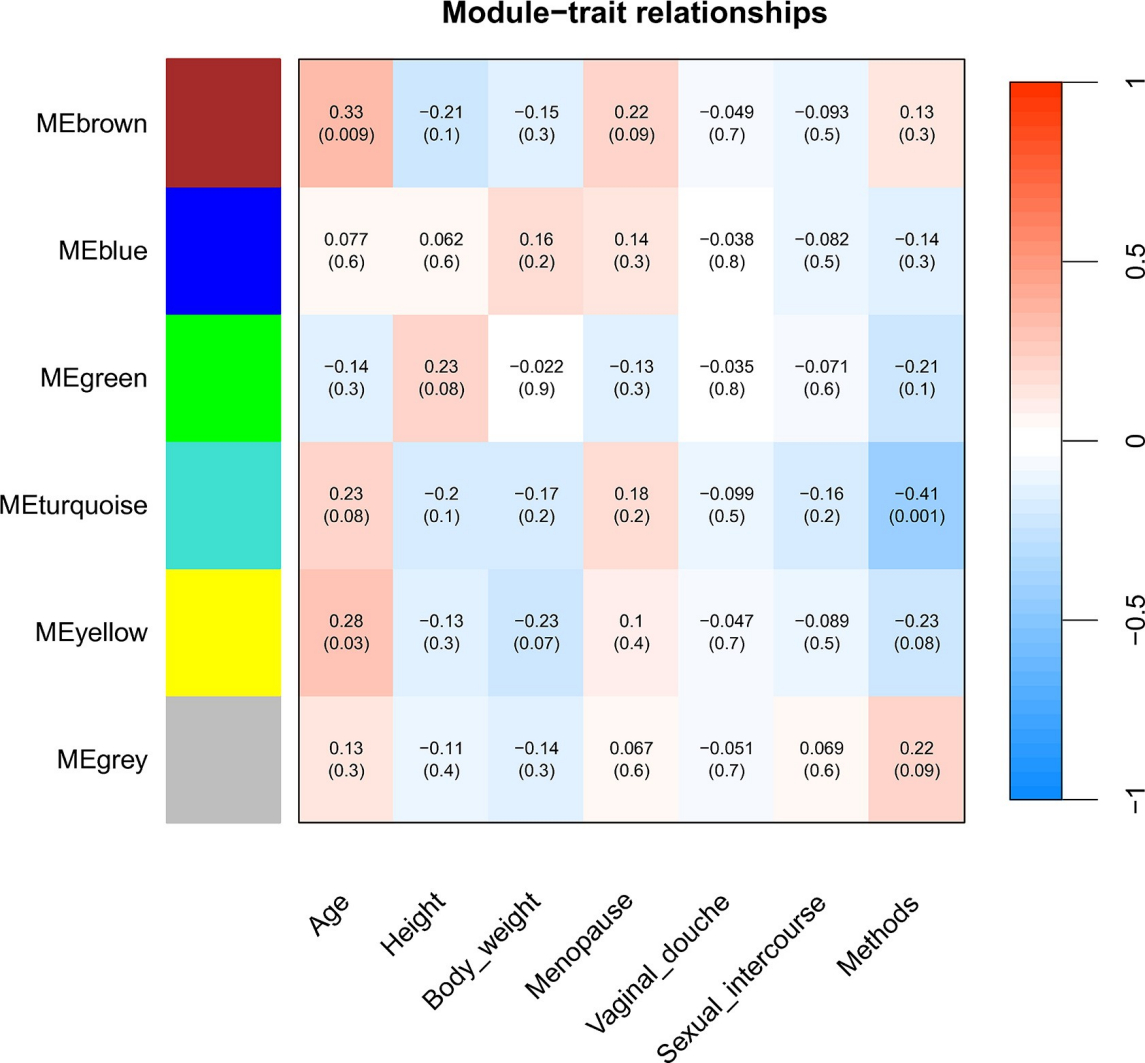
WGCNA analysis of LBC and SWAB microbiomes at the genus level. Relevance of module eigen microbiome with clinical traits. The number in each cell was the correlation coefficient between each module and clinical traits, and the number below was the corresponding p-value. The depth of color indicated the value of the correlation; red represented the positive correlation, and blue represented the negative correlation.

WGCNA identified six modules based on abundance data, establishing interactions with six clinical data and sampling methods (Fig 6). The MEturquoise group showed a correlation of −0.41 and a significance of 0.001, indicating a significant difference depending on the method. There was a correlation of 0.33 and significance of 0.009 between age and the MEbrown group and a correlation of 0.28 and significance of 0.03 between age and the MEyellow group. However, the relationship between the six modules and other clinical information was not significant, using 0.05 as the standard for significance.

## Discussion

Our study used a comprehensive methodology that integrated diversity metrics, taxonomic classification, network analysis, and machine learning. This approach allowed us to gain a multifaceted understanding of the cervical microbiome based on the sampling methods. Integrating LBC with cervical microbiome testing is a significant challenge in gynecological diagnostics [14].

The Shannon index measures the richness and evenness of species in a community, whereas Simpson index measures only species dominance. Shannon index, which is a measure of species richness and evenness, was higher in the LBC group (mean = 3.72) than in the SWAB group (mean = 3.18). This difference was statistically significant (p = 0.032), suggesting that the LBC group harbored a more diverse microbial community. The higher Shannon index in the LBC group suggests greater diversity, possibly influenced by a broader range of microbial species and a more even distribution than the SWAB group [26]. However, the lack of significant differences in the Simpson index indicates that, despite variations in overall diversity, the dominance or abundance of specific taxa may not differ substantially between the two sampling methods [27]. This could imply that while the LBC group may harbor a more diverse set of microbes, the core composition and dominance of certain species remain relatively consistent between the LBC and SWAB samples. Conversely, beta diversity evaluates the differences in diversity between two or more samples or treatment groups, emphasizing the dissimilarity in the overall microbial composition. We conducted beta diversity analysis using the Bray-Curtis method and PCoA to compare the microbial communities between the LBC and SWAB groups. The non-significant result (p = 0.99, as determined using PERMANOVA) in beta diversity suggests that the differences observed in the alpha diversity metrics between LBC and SWAB showed a statistically non-significant impact on the overall composition or structure of the cervical microbial community [28].

The taxonomic classification of the microbiome data obtained from cervical LBC and swab SWAB samples revealed a consistent microbial signature across both sampling techniques. In the order of abundance, the bacteria identified were *Lactobacillus*, *Gardnerella*, *Bifidobacterium*, *Prevotella*, and *Streptococcus* spp. The presence of *Lactobacillus* spp. in the cervix is consistent with a previous study on the vaginal microbiota, as it is the most common and beneficial microorganism found in the healthy human vagina [29]. The presence of *Gardnerella*, *Bifidobacterium*, *Prevotella*, and *Streptococcus* spp. in the cervix may indicate a condition known as bacterial vaginosis [30]. This condition is characterized by a reduction or sharp decline in the total number of *Lactobacillus* spp. and a corresponding significant increase in the concentration of anaerobic microbes. Bacterial vaginosis is a common disorder of the vaginal microbiota in women of reproductive age. It is associated with negative gynecological and obstetric consequences, including sexually transmitted infections, pelvic inflammatory diseases, and preterm birth [31]. Therefore, these bacteria serve as a promising marker for assessing the precision and reliability of microbial community characterization in the cervical environment. In this study, a strong correlation coefficient (> 0.9 was observed for 25 samples, indicating a consistently stable microbial signature across both the LBC and SWAB sampling techniques. The high correlation indicates a strong agreement in the microbial composition detected using the two sampling methods. A comprehensive analysis was conducted on the four individuals with the highest sequencing quality, focusing on a correlation analysis of the top 10 microorganisms in terms of abundance. The high correlation coefficients observed for these individuals further reinforced the consistency of the microbial signature across different sampling methods.

Hierarchical clustering based on these microorganisms revealed a pattern in which the similarity between samples from the same individual was more pronounced than the differences arising from diverse sampling methods. This indicates a distinct microbial pattern unique to each individual. Further analysis confirmed that the 20 microorganisms, whose abundances varied depending on the sampling method, did not represent the overall population but existed only in one sample. In other words, the differences in the observed abundance based on this method were not statistically significant when considering a broader microbial community. Functional enrichment analysis revealed two significant KEGG pathways within the cervical microbiome: arachidonic acid and alpha-linolenic acid metabolism. Although statistically significant, the small mean proportion values associated with these pathways urge caution when attributing functional distinctions exclusively to the sampling methods.

Co-occurrence pattern analysis within the cervical microbiome revealed a network structure characterized by a positive correlation among bacterial taxa. Statistical analysis reveal non-significant results regarding the characteristics of the network structure, such as the average number of connected edges per node, number of nodes, network density, and heterogeneity. These findings suggest a consistency in the structural characteristics of the microbial networks generated by LBC and SWAB [32]. Taxonomic classification and subsequent cluster analysis of the cervical microbiome samples identified the top five microorganisms within the network. *Lactobacilli*, one of the most abundant bacteria, consistently exhibited clustering characteristics across both sampling methods, displaying distinct abundance patterns compared with other microorganisms. The consistent clustering characteristics of Lactobacilli across different sampling methods suggest that this genus may play a crucial role in shaping the cervical microbial community, regardless of the sampling method. This consistency is especially important because *Lactobacilli* are crucial for vaginal health, playing a key role in maintaining acidic pH levels and preventing the proliferation of pathogenic microorganisms [33]. The consistent clustering characteristics of *Lactobacilli* across different sampling techniques indicate the robustness of their prevalence, reinforcing their importance as key indicators of cervical health [34]. The consistency in the network structures further supports the concept that the fundamental microbial community remains preserved regardless of the specific sampling method.

This study applied supervised machine learning methods to assign labels to distinct groups based on the sampling method, thereby enabling the examination of the explanatory power of the selected variables [35]. The explanatory powers of the first and second variables, as determined through the maximum distance method, were 7% and 10%, respectively. Identifying a clear boundary separating the two methods with 35 samples positioned at this interface suggests that samples near the border exhibit characteristics that make assigning one sampling method unequivocally over the other challenging. The increase in error rates as more main components were included during feature selection and classification offers valuable insights into the complexities of distinguishing between LBC and SWAB. The initial error rate of 36% indicates that, initially, the selected features derived from differences between the two sampling methods resulted in misclassifications in a significant portion of the dataset. As the analysis progressed, the subsequent error rates of 39%, 43%, and finally 47% indicated that the selected features may not reliably distinguish between sampling methods, even when derived from differences between the two methods.

The WGCNA conducted on the cervical microbiome data revealed correlations between microbial clusters and clinical parameters. Identifying six distinct modules based on microbial abundance provides a comprehensive understanding of the complex relationships within the cervical microbiome. The MEturquoise group showed a significant correlation of −0.41 with the difference in the sampling methods. However, this group did not show significant correlations with other clinical data. This suggests that the sampling method influenced the formation of the MEturquoise microbial community but had a low correlation with the clinical data used for diagnosis. Moreover, the absence of significant correlations between the remaining five differences in the methods suggests that the microbial populations are not influenced by the sampling methods. A positive correlation of 0.33 was observed between age and the MEbrown group, as well as a correlation of 0.28 between age and the MEyellow group. Although age can affect the cervical microbiome, the absence of significant correlations with other clinical data emphasizes the necessity for more focused research to unravel the complex influences on microbial shift within the cervix [36].

In summary, our comprehensive approach, which combines diversity metrics, taxonomic classification, network analysis, and machine learning, provides a multifaceted understanding of the cervical microbiome. Integrating LBC with cervical microbiome testing is a significant challenge in gynecological diagnostics. This dual diagnostic approach enables the examination of cellular abnormalities and allows for comprehensive profiling of the cervical microbiome, which is increasingly recognized for its role in cervical health and disease. Using residual LBC as a biospecimen source provides an opportunity to conduct large-scale microbiome studies. The availability of these samples from millions of patients worldwide represents a valuable resource for identifying microbial biomarkers of gynecological diseases, including cervical cancer. The potential to utilize these easily accessible samples could revolutionize the regular screening of women for gynecological cancers. Furthermore, the ability to conduct simultaneous cytology and microbiome testing could provide a more comprehensive understanding of cervical health. This could reveal correlations between cellular abnormalities and specific changes in the microbiome, providing new insights into the pathogenesis of cervical diseases. This discovery can potentially facilitate the development of novel diagnostic and therapeutic strategies, such as microbiome-based biomarkers or probiotic treatments for cervical health.

## Supporting information

S1 Table The result of filtered features. (XLSX)

S1 Fig Diversity analysis of LBC and SWAB microbial composition. Bray-Curtis distances methods of bacterial 16S rRNA genes. Correlation between microorganisms according to the same intra-individual sampling method was 0.9 or higher and was marked as having become a cluster (blue), and conversely, if the correlation was less than 0.9, it was marked as not being a cluster (orange).
(TIF)

S2 Fig Correlation between sample methods of Viome8 microbiome composition at the genus level. (a) Using a 2-group comparison, the correlation between compositions at the species level was found to be 0.999. (b) The 16 identified features were selected, and their significance for presence or absence was examined. (c) Lactobacillus was the most dominant in both sampling methods.
(TIF)
